# Biphenyl Wrinkled Mesoporous Silica Nanoparticles for pH-Responsive Doxorubicin Drug Delivery

**DOI:** 10.3390/ma13081998

**Published:** 2020-04-24

**Authors:** Jason Lin, Chuanqi Peng, Sanjana Ravi, A. K. M. Nur Alam Siddiki, Jie Zheng, Kenneth J. Balkus

**Affiliations:** Department of Chemistry and Biochemistry of the University of Texas at Dallas, 800 W Campbell Rd., Richardson, TX 75080, USA; jxl140030@utdallas.edu (J.L.); cxp141630@utdallas.edu (C.P.); Sanjana.Ravi@utdallas.edu (S.R.); siddiki@utdallas.edu (A.K.M.N.A.S.); jiezheng@utdallas.edu (J.Z.)

**Keywords:** wrinkled mesoporous silica, drug delivery, pH selective, doxorubicin

## Abstract

Biphenyl wrinkled mesoporous silica nanoparticles with controlled particle size and high surface area were evaluated for the storage and delivery of doxorubicin. The average particle size and surface area were ~70 nm and ~1100 m^2^/g. The doxorubicin loading efficiency was 38.2 ± 1.5 (w/w)% and the release was pH dependent. The breast cancer cell line, MCF-7 (Michigan Cancer Foundation-7) was used for the in vitro drug release study. The cytotoxicity of doxorubicin-loaded nanoparticles was significantly higher than free doxorubicin. Fluorescence images showed biphenyl wrinkled mesoporous silica (BPWS) uptake by the MCF-7 cells. The biphenyl bridged wrinkled silica nanoparticles appear promising for hydrophobic drug loading and delivery.

## 1. Introduction

**Figure 1:**
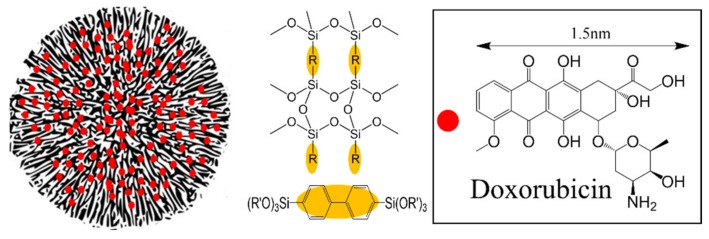
Biphenyl bridged wrinkled silica loaded with doxorubicin.

Nanomedicine [1,2,3,4] and associated drug delivery systems are dependent on both the particle size and drug loading efficiency. The role of porosity is less studied but important for drug loading. Porous materials such as mesoporous silica nanoparticles (Mobil Composition of Matter, MCM-41 and Santa Barbara, SBA-15) are attractive for biomedical applications because of their high surface area, low cytotoxicity, good biocompatibility, tunable morphologies, and ease of functionalization through basic siloxane chemistry [5,6,7,8,9,10,11]. For example, Lozano et al. reported the uptake and delivery of L-tryptophan using SBA-15 for bone repair [12]. Kim et al. reported ultrasound triggered MCM-41 type mesoporous silica for controlled ibuprofen release [13]. Lu et al. demonstrated that mesoporous silica nanoparticles (MSNs) were biocompatible and preferentially accumulate in tumors due to abnormal tumor blood vessels [14]. Lai et al. reported MCM-41 using cadmium sulfide as a gatekeeper for the controlled release of vancomycin and adenosine triphosphate [15]. MSNs with phosphorylated lipids have also been used for the controlled release of fluorescein [16]. MSNs loaded with both aluminum chloride phthalocyanine and cisplatin have been studied for cancer treatment [17]. Di Pasqua et al. reported using neutron-activatable Holmium-containing MSNs for radiotherapy. The anticancer drug carboplatin-loaded mesoporous silica was also used as a radiosensitizer [18,19]. The mesoporous silica DAM-1 (Dallas Amorphous Material-1) templated by Vitamin E-TPGS (α-tocopheryl polyethylene glycol 1000 succinate) was used to deliver hydrophobic drugs [20]. While, MCM-41 and SBA-15 type materials have been the most widely studied mesoporous materials for drug delivery, the 1-D pore structure of these materials could limit drug storage and release. In contrast, spherical wrinkled mesoporous silica (WMS), also referred to as fibrous mesoporous silica, such as KCC-1, has an even higher surface area and more accessible pores [21] than MCM-41 or SBA-15. There have been comparatively fewer reports of drug delivery using WMS type materials. For example, Yang et al. reported fibrous mesoporous silica on Fe_3_O_4_ for doxorubicin drug delivery [22,23]. We first reported wrinkled periodic mesoporous organosilica (PMOs) nanoparticles for hydrophobic anticancer drug Paclitaxel delivery [24]. Periodic mesoporous organosilicas (PMOs) comprise a sub class of mesoporous silica materials, where the structure contains both organic and inorganic components as shown in Figure 1. In the case where R is phenyl, the PMOs not only showed a higher loading of Paclitaxel but also showed a slower and sustained release compared to mesoporous silica.

In this study, we have extended our work with the PMOs by incorporating a biphenyl group (Figure 1) and employing this wrinkled mesoporous silica as a drug delivery vehicle. 4,4′-bis(triethoxysilyl)-1,1′-biphenyl (BP) and tetraethyl orthosilicate (TEOS) were used as the silica precursors with hexadecylpyridinium bromide (CPB) as the template to form the high surface area biphenyl wrinkled mesoporous silica (BPWS). The hydrophobic anticancer drug, doxorubicin (DOX) shown in Figure 1, is ~1.5 nm in diameter and readily absorbed in the mesopores lined with the biphenyl groups [25]. In vitro results showed that the DOX-loaded BPWS (DOX-BPWS) exhibited cytotoxicity that was significantly higher than the free DOX with negligible cytotoxicity from the BPWS itself. The effective delivery of DOX from the BPWS may lead to a variety of future applications for periodic mesoporous organosilicas. 

## 2. Materials and Methods 

### 2.1. Experimental Materials

Urea was purchased from (Paris, KY, USA). Hexadecylpyridinium bromide (≥97%), isopropyl alcohol (≥99.7%), 3-(aminopropyl) trimethoxylsilane and 4,4′-bis(triethoxysilyl)-1,1′-biphenyl were purchased from Sigma Aldrich (St. Louis, MO, USA). Cyclohexane was purchased from EMD Millipore Corporation (Burlington, MA, USA). Tetraethyl orthosilicate (TEOS) (98%), NHS-Fluorescein (FITC), dimethyl sulfoxide (DMSO), 3-(4,5-dimethylthiazol-2-yl)-2,5-diphenyltetrazolium bromide (MTT), hydrochloric acid (HCl) and ethanol 95% (EtOH) were purchased from Fisher Scientific (Waltham, MA, USA). Doxorubicin hydrochloride (DOX·HCl) was purchased from Tecoland Corporation (Irvine, CA, USA). The human breast cancer cell line MCF-7 was purchased from ATCC (Manassas, VA, USA). All chemicals were used without further purification.

### 2.2. Characterization

The biphenyl wrinkled silica nanoparticles were analyzed by transmission electron microscopy (TEM) using an analytical JEOL 2100 microscope (Tokyo, Japan) with an accelerating voltage of 200 kV. Scanning electron microscopy (SEM) images were obtained using a Zeiss-LEO model 1530 scanning electron microscope (Monument, CO, USA). Nitrogen gas adsorption analysis used a Quantachrome AS1 autosorb (Graz, Austria). Thermogravimetric analysis (TGA) analysis was conducted using a TA Instruments SDT Q600. PTI QuantaMasterTM 30 Fluorescence Spectrophotometer (Birmingham, NJ, USA) was used to measure the fluorescence of BPWS and DOX. Ultraviolet Visible (UV–Vis) spectroscopy of doxorubicin was carried out using a Shimadzu UV-1601PC spectrophotometer (Kyoto, Japan). The fluorescence microscopic images were obtained using an Olympus IX71 microscope (Tokyo, Japan) with a PhotonMax 512B CCD camera (Princeton Instrument).

### 2.3. Experimental Methods

#### 2.3.1. Synthesis of Biphenyl Wrinkled Mesoporous Silica (BPWS)

BPWS was synthesized by dissolving 0.3 g of urea and 0.5 g of CPB in 15 mL of deionized (DI) H_2_O in a 100 mL round bottom flask (~pH 6.7). Next, 0.23 mL of isopropyl alcohol was added and stirred for 30 min at RT. Then, 15 mL of cyclohexane was added and stirred until all the CPB surfactant was dissolved (~30 min). Then, 0.29 mL (0.6 mmol) of 4,4′-bis(triethoxysilyl)-1,1′-biphenyl and 0.55 mL (2.4 mmol) of TEOS were added. After 30 min, the mixture was refluxed 24 h at 70 °C (~pH 7.5). The solution was cooled to RT and centrifuged (6000 rpm, 5 min) and washed three times with 95% ethanol. The sample was re-dispersed in 60 mL of 95% ethanol and 1 mL of 12M HCl and refluxed 24 h at 70 °C to extract the CPB surfactant. After centrifugation, the sample was dried at 80 °C overnight. The reaction pH was ~6.7-6.9 before heating, and ~7.5–7.8 after 24 h at 70 °C.

#### 2.3.2. Doxorubicin-Loaded BPWS (DOX-BPWS) and pH-Dependent Release Study

Doxorubicin is both light and temperature sensitive and therefore, should be stored at 0 °C before use. All experiments were performed under light controlled conditions. BPWS (10 mg) was dispersed in 10 mL DI H_2_O (solution A) in a 22 mL vial. Doxorubicin (10 mg) was dissolved in 10 mL DI H_2_O (solution B). 1 mL of solution A and 1mL of solution B were combined and vortexed for 24 h at RT. DOX-BPWS was collected by centrifugation and the supernatant was analyzed by UV-Vis to calculate the amount of drug loaded. The sample was further washed with DI H_2_O 3 times. The collected DOX-BPWS was dispersed in 10 mL phosphate buffer solution (PBS). 1 mL DOX-BPWS PBS solution was further diluted 10 times in two different pH (7.4 and 5.5) and kept at 37 °C. Both samples were centrifuged (12,000 rpm, 10 min) and replaced with fresh 10 mL PBS after 1, 2, 3, 6, 9 h. The supernatant was collected and analyzed by fluorescence spectroscopy to determine the concentration of the drug released. All experiments were reproduced three times.

#### 2.3.3. In Vitro Cytotoxicity Study

For the cytotoxicity study, MCF-7 cells (1000 cells/well) were seeded in 96-well plates and cultured in Dulbecco’s modified eagle medium (DMEM) with supplements (10% fetal bovine serum, FBS, 1% penicillin-streptomycin) at 37 °C in a humidified atmosphere containing 5% CO_2_ overnight. The cells were then exposed to DOX-BPWS or free DOX (containing DOX at different concentrations ranging from 0.1 to 50 µM) in DMEM with supplements, whereas blank DMEM with supplements was used as a control. The cytotoxicity of the BPWS vehicle was also evaluated. The BPWS concentration was equivalent to DOX-BPWS. After incubation at 37 °C for 72 h, the culture media were replaced with MTT (3-(4,5-dimethylthiazol-2-yl)-2,5-diphenyltetrazolium bromide, 0.5 mg/mL). After further incubation at 37 °C for 3 h, the solution was removed and refilled with dimethyl sulfoxide (DMSO) and analyzed by a BMG LABTECH FLUOstar OPTIMA microplate reader to obtain the IC_50_ (half maximal inhibitory concentration) of DOX. These experiments were reproduced three times.

#### 2.3.4. Synthesis of FITC Labeled Biphenyl Wrinkled Mesoporous Silica (FITC-BPWS)

BPWS (1 g) was dispersed in 10 mL DI H_2_O in a 50 mL centrifuge tube and 0.1 mL of 3-(aminopropyl) trimethoxylsilane was added to the solution. After 10 min of sonication to disperse the BPWS, 1 mL 1M NaOH was added to the solution and sonicated for another 30 min. The sample was centrifuged (6000 rpm, 5 min), collected and re-dispersed in 100 mL DI H_2_O in a 250 mL round bottom flask. 1 mg/mL of sample was transferred to a 22 mL vial and was diluted with 9 mL DI H_2_O. FITC (1 mg) was added to the vial and vortexed for 24 h. The FITC-BPWS was centrifuged (6000 rpm, 5 min), collect and dispersed in 1 mL PBS solution.

#### 2.3.5. In Vitro Cellular Uptake and Imaging

For the fluorescence cellular imaging, the cancer cells (5000 cells/dish) were seeded in culture dishes with 1.5 mL of DMEM with supplements. After culture for 24 h, the cells were exposed to 2 µg/mL of FITC-BPWS or blank PBS in DMEM with supplements at 37 °C at different time intervals over 24 h. The cells were washed, fixed, and stained with 4% formaldehyde and 4′,6-diamidino-2-phenylindole (DAPI) for 10 min before fluorescence microscopic imaging. Fluorescence images of both FITC-BPWS treated cells and PBS-treated cells were captured using a fluorescence microscope. The fluorescence microscopic images were obtained using an Olympus IX71 microscope with a PhotonMax 512B CCD camera (Princeton Instrument). Excitation: 480 ± 20 nm, emission: 535 ± 20 nm. 

## 3. Results and Discussion

### 3.1. Material Physical Properties

**Figure 2:**
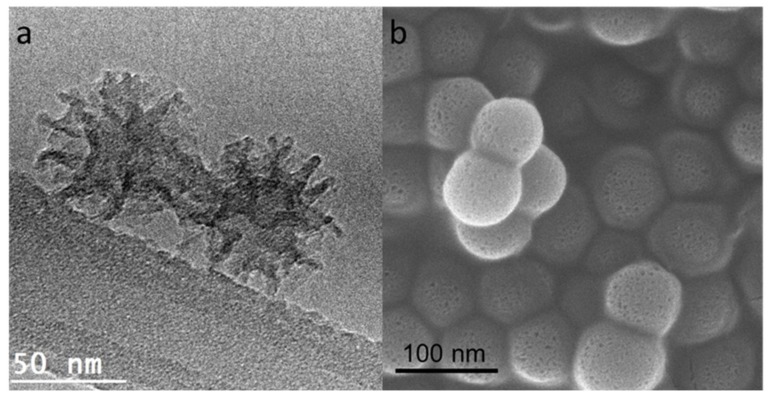
(**a**) Transmission electron microscopy (TEM) and (**b**) scanning electron microscopy (SEM) images of biphenyl wrinkled silica.

The morphology and the particle size were analyzed by Transmission electron microscopy (TEM) and scanning electron microscopy (SEM) as shown in Figure 2. The TEM image (Figure 2a) shows the PMO, sphere like nanoparticles have a dendrimer-like wrinkled structure. The SEM image (Figure 2b) shows a spherical morphology. These spherical wrinkled structures provide easy drug loading and release. The average particle size of BPWS was 71.6 ± 7.1 nm based on the histogram in Figure S1a. The dynamic light scattering (DLS) hydrodynamic diameter was 81.7 ± 10.8 nm (Figure S1b). Compared to our previous work using phenyl silane, the BPWS possess a smaller particle size [24]. It has been reported that a nanoparticle size of ~50 nm may result in higher tumor uptake by the enhanced permeability and retention (EPR) effect [26]. For example, Lu et al. reported that MSNs ~50 nm in diameter had the highest loading in HeLa cells [27].

The Nitrogen gas adsorption–desorption isotherm shown in the Figure S2 exhibits a type IV isotherm with a H3 type hysteresis. The Brunauer–Emmett–Teller (BET) surface area of the BPWS nanoparticles was 1167 m^2^/g. The relatively high BPWS surface area should favor drug loading [28].

TGA indicates that the BPWS nanoparticles are stable up to 500 °C. The 5% weight loss observed before 100 °C is from water. Although the BPWS contained hydrophobic biphenyl moieties, the Si-O-Si silicate framework still some hydrophilic character. The nature of the BPWS hydrophilic and hydrophobic domains will depend on the loading of organosilane. The starting mole ratio of BP and TEOS is 1:4 such that the BPWS should contain 36% by weight biphenyl after surfactant removal (Supporting Information). A 40% weight loss was observed around 500–600 °C indicating the decomposition of biphenyl groups (Figure S3).

### 3.2. Material In Vitro Studies

**Figure 3:**
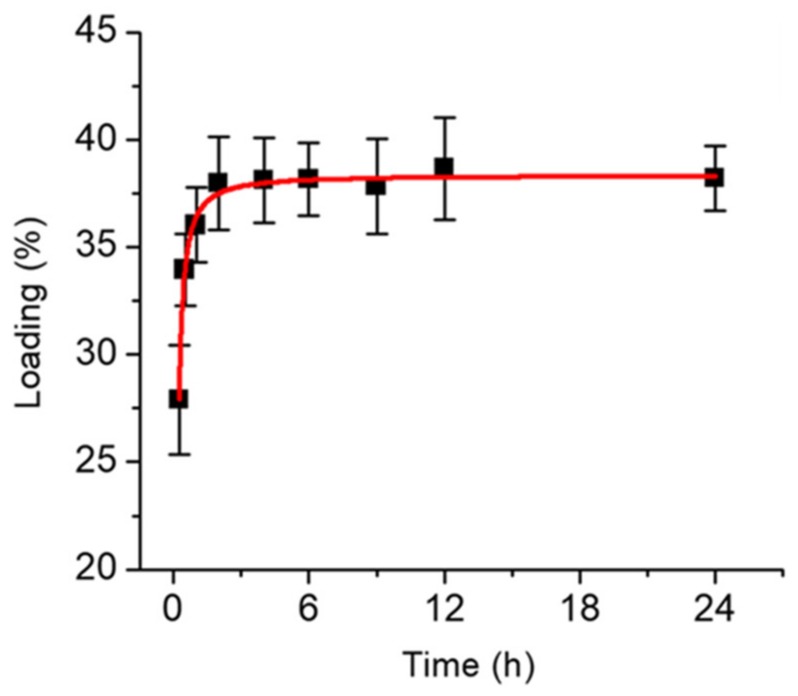
A plot of doxorubicin loading versus time for biphenyl wrinkled mesoporous silica (BPWS) nanoparticles.

Doxorubicin is one of the most commonly used hydrophobic cancer drugs, where the clinical dosage for humans is 5 mg/kg body weight [29,30,31,32]. Doxorubicin was loaded in the BPWS nanoparticles and the amount adsorbed was determined by UV-VIS spectroscopy (Figure S4). Figure 3 shows the maximum drug loading was 38.2 ± 1.5 (w/w)% which indicates that for every 1 mg of BPWS 0.38 mg of DOX was loaded. The largest DOX uptake by the BPWS occurred in the first hour and reached a maximum loading after 3 h. This high drug uptake may reflect a strong interaction between the biphenyl moieties and the hydrophobic doxorubicin as well as the high surface area of the BPWS vehicle. Croissant et al. reported a 32 wt.% loading of DOX in protein-gold cluster-capped mesoporous silica nanoparticles (~160 nm) [33]. Doxorubicin was also loaded on polyvinyl alcohol (PVA) coated iron oxide nanoparticles with only a 5.8 wt.% loading [34]. Thus the hydrophobic and mesoporous nature of BPWS makes it a promising vehicle for doxorubicin storage and delivery.

**Figure 4:**
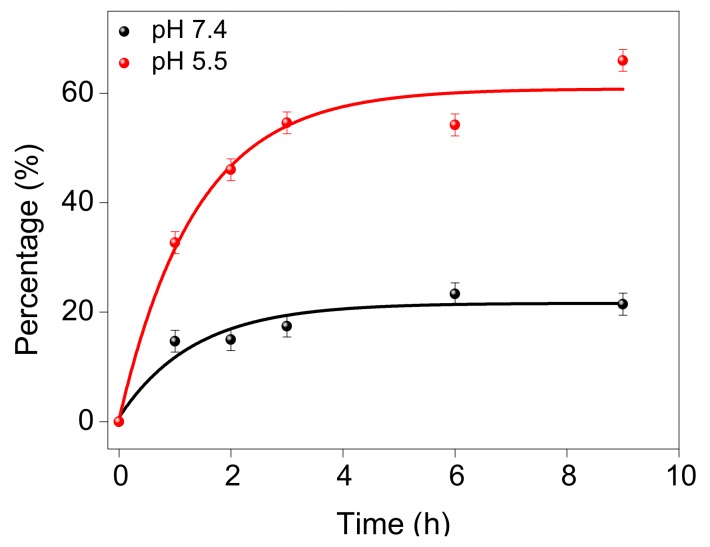
A plot of percentage of doxorubicin (DOX) released versus time at pH 5.5 and pH 7.4.

DOX-BPWS was dispersed at two different pH conditions 7.4 and 5.5 for the drug release tests. The concentration of released drugs was measured by fluorescence (Figure S5). Figure 4 shows that only 20% (0.13 µmole) of the DOX was released at pH 7.4, while 60% was released at pH 5.4. These results imply the DOX-BPWS will be stable under physiological conditions (pH 7.4) but the DOX will largely be released in the intracellular acidic regions (pH 5) such as endosomes and lysosomes. The pH-responsive release may be attributed to the protonation of the NH_2_ groups on DOX at lower pH. Thus, the positive charge will reduce the hydrophobic interaction between BPWS and DOX [35]. Hakeem et al. also reported a pH response from polyaspartic acid decorated mesoporous silica nanoparticles but with only a 6.5 wt.% DOX loading efficiency [36]. In this case, 10% DOX release was observed at pH 7.4 and 56% release at pH 5.5 [36]. Du et al. reported a pH-sensitive polymer for DOX delivery with a similar 8.3 wt.% loading and a 20% release at pH 7.4 versus 70% release at pH 5.0 [37].

**Figure 5:**
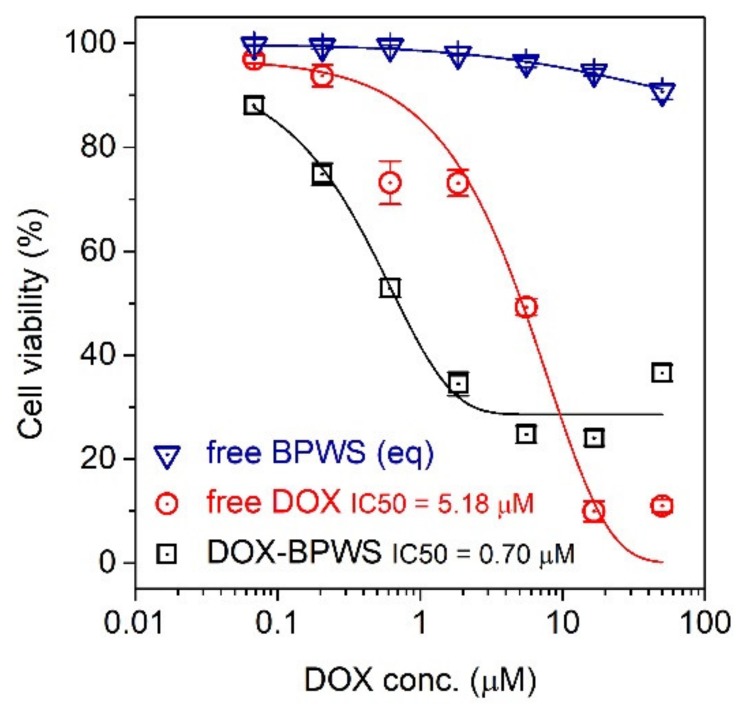
A plot of cell viability versus DOX concentration for 3-(4,5-dimethylthiazol-2-yl)-2,5-diphenyltetrazolium bromide (MTT) assays of MCF-7 Cells with doxorubicin, DOX-loaded BPWS and free BPWS as well as the IC_50_ after 72 h.

To verify that the released DOX was pharmacologically active, an in vitro MTT assay was carried out utilizing an MCF-7 cell line. The cytotoxicity of the free BPWS, free DOX and DOX-BPWS against the MCF-7 cells was evaluated. The cells were exposed to the drug for 72 h, and then the cell viability was characterized by an MTT assay. Figure 5 shows that IC_50_ (half maximal inhibitory concentration) of the DOX and DOX-BPWS were 5.18 µM and 0.70 µM, respectively. The control with drug free BPWS only showed no obvious cytotoxicity. Thus, the cytotoxicity of the DOX-BPWS was from the released DOX, not the BPWS vehicle. The DOX-BPWS toxicity was sevenfold stronger compared to the free DOX after 72 h. This can be compared to a report of doxorubicin-loaded mesoporous silica particles (~200 nm) modified with amine-terminated polyamidoamine dendrimers that exhibited an IC_50_ of 1.07 µM with an ~40% (w/w) DOX loading [38]. The DOX-BPWS showed a higher cytotoxicity with a smaller nanoparticle size. The increased cytotoxicity of DOX-BPWS versus free DOX may be due to a pathology difference. Free DOX is known to enter the cells through passive diffusion; whereas, DOX-BPWS maybe entered the cells through endocytosis. These results indicate that the BPWS may be an effective and safe drug delivery carrier for future in vivo studies. 

**Figure 6:**
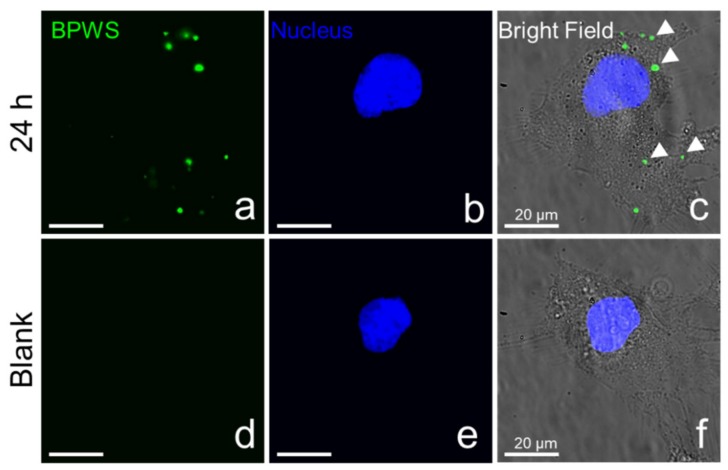
Fluorescence images of cancer cell permeability and intracellular uptake from the (**a**) NHS-Fluorescein (FITC) fluorescence signal, (**b**) 4′,6-diamidino-2-phenylindole (DAPI) fluorescence signal, and (**c**) bright field merged image from FITC labeled biphenyl wrinkled mesoporous silica (FITC-BPWS) treated cells. Images (**d**) FITC fluorescence signal, (**e**) DAPI fluorescence signal, and (**f**) bright field merged image are from phosphate buffer solution (PBS)-treated cells.

To get a better understanding of the intracellular distribution of BPWS and the released drug, fluorescence microscope images were taken for both DOX and DOX-loaded BPWS (Figure S6). These images support the MTT assay that shows the DOX was taken up by the cell. However, the DOX-BPWS could not be imaged because the emission of the biphenyl moieties was at 380 nm which is below the shortest fluorometer wavelength. Therefore, BPWS was surface modified with amine groups and conjugated with FITC which emits at 535 nm (Figure S7). The cell nucleus was stained with DAPI 10 min prior to fluorescence imaging. As shown in Figure 6, the FITC fluorescence signal can clearly be observed in FITC-BPWS treated cells (Figure 6a). Overlapping the FITC fluorescence signal with the DAPI fluorescence signal (Figure 6b) as well as the bright field image of the cells (Figure 6c), show that the FITC-BPWS is taken up by MCF-7 cells and is located in the cytosol of MCF-7 cells. No fluorescence signal from the FITC channel was observed in the PBS-treated control blank cells (Figure 6d). This eliminates any interference from autofluorescence. Additional cell images are shown in Figure S8.

## 4. Conclusions

Porous materials such as wrinkled mesoporous silica have a promising future in nanomedicine. The porosity, particle size and surface functionality can be modified to facilitate the uptake of the anticancer drugs such as DOX. The particle size is particularly important for cell permeability, where nanoparticles in the range of ~50 nm maybe optimal. Additionally, the wrinkled mesoporous silica nanoparticles can be made fluorescent for imaging. The MTT assay of MCF-7 shows that the cytotoxicity of BPWS vehicle is negligible, while the cytotoxicity of DOX-BPWS is seven times higher than for free DOX. The flexible design of PMO frameworks such as BPWS may lead to theranostic nanoparticles for a variety of medical applications.

## Supplementary Materials

The following are available online at https://www.mdpi.com/1996-1944/13/8/1998/s1, Figure S1: BPWS size distribution histogram from TEM images and DLS, Figure S2: Nitrogen gas adsorption–desorption isotherms of biphenyl wrinkled silica, Figure S3: TGA weight loss of BPWS and BPWS before and after surfactant extraction, Figure S4: UV-VIS spectra at various concentrations, Figure S5: Fluorescence spectra of DOX-BPWS released at different pHs, Figure S6: Fluorescence microscope images (DOX emission) of MCF-7 breast cancer cells after 24 h, Figure S7: TEM image of FITC-BPWS, Figure S8: Additional fluorescence images of cancer cellular uptake.
