# Supplementary material for: Biphenyl Wrinkled Mesoporous Silica Nanoparticles for pH-Responsive Doxorubicin Drug Delivery
Source: Materials (Basel). 2020 Apr 24;13(8):1998. doi: 10.3390/ma13081998 (PMC7215304; doi:10.3390/ma13081998)
Supplement: Supplementary file 1 [file materials-13-01998-s001.pdf]

materials

Supplementary information

Article

Biphenyl Wrinkled Mesoporous Silica Nanoparticles for pH-Responsive Doxorubicin Drug Delivery

Jason Lin, Chuanqi Peng, Sanjana Ravi, A K M Nur Alam Siddiki, Jie Zheng and Kenneth J. Balkus Jr. *

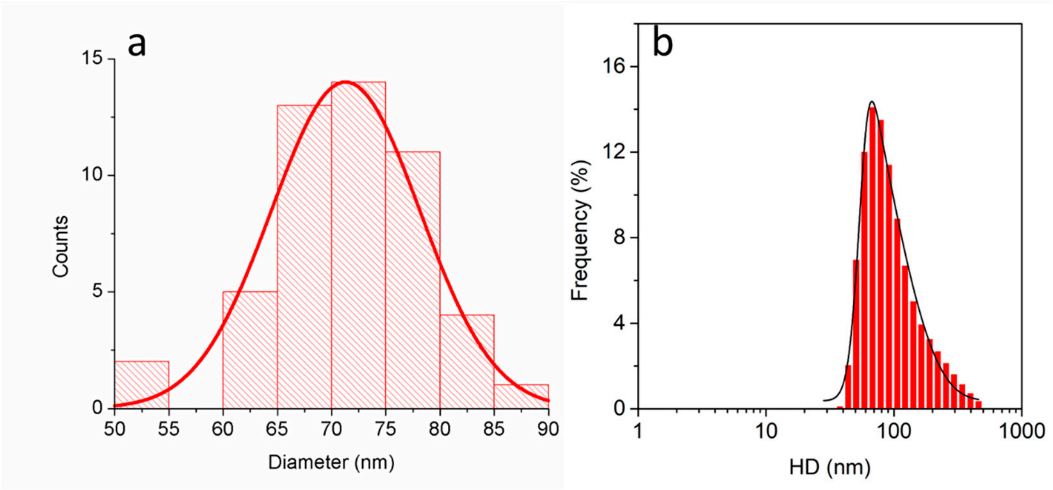

Figure S1. BPWS size distribution histogram from (**a**) TEM images (size: 71.6 ± 7.1 nm), and (**b**) DLS hydrodynamic diameter (size: 81.7 ± 10.8 nm).

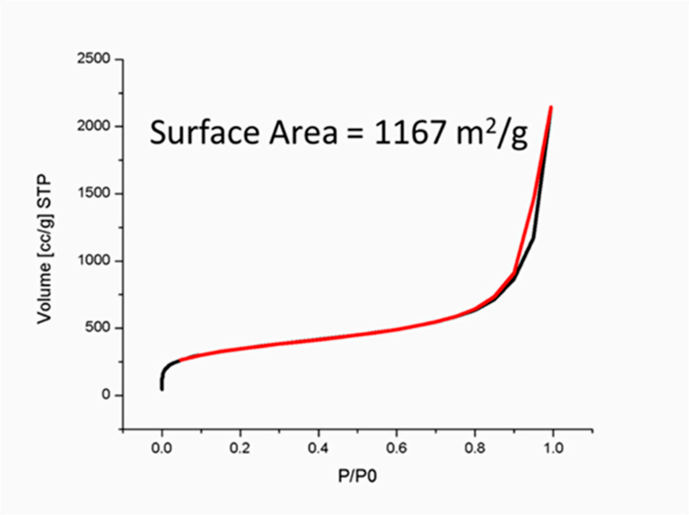

Figure S2. Nitrogen gas adsorption–desorption isotherms of biphenyl wrinkled silica.

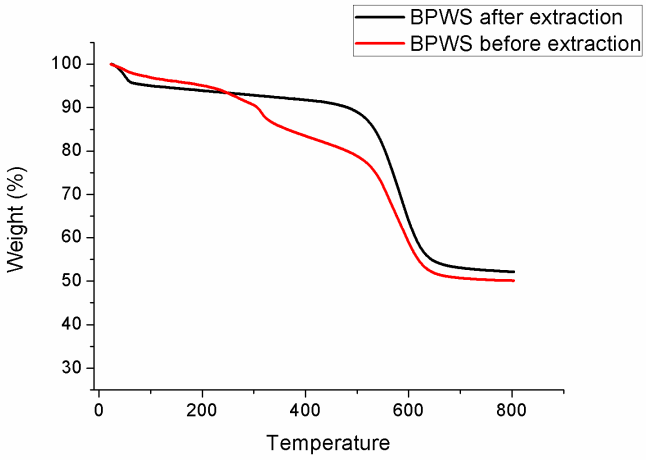

Figure S3. TGA weight loss of BPWS and BPWS before and after surfactant extraction.

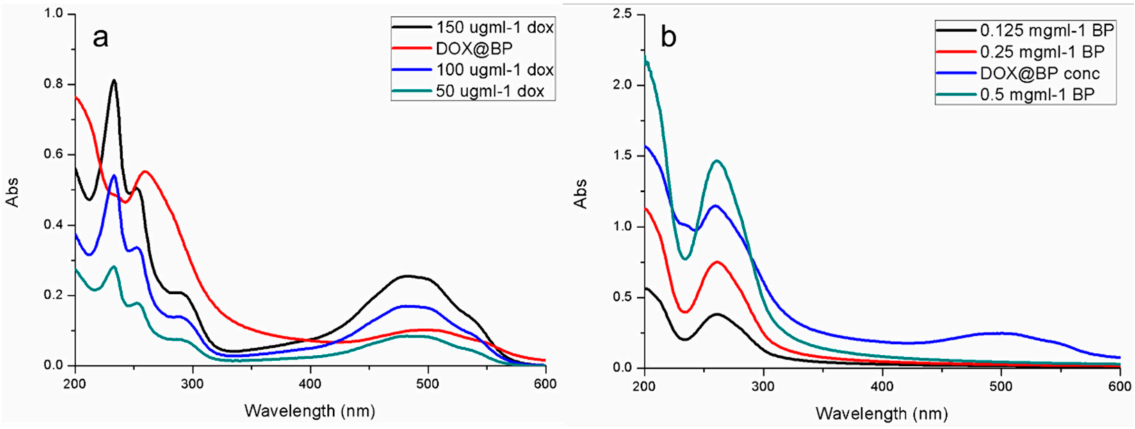

Figure S4. UV-Vis spectra of (**a**) DOX and (**b**) BP at different concentrations.

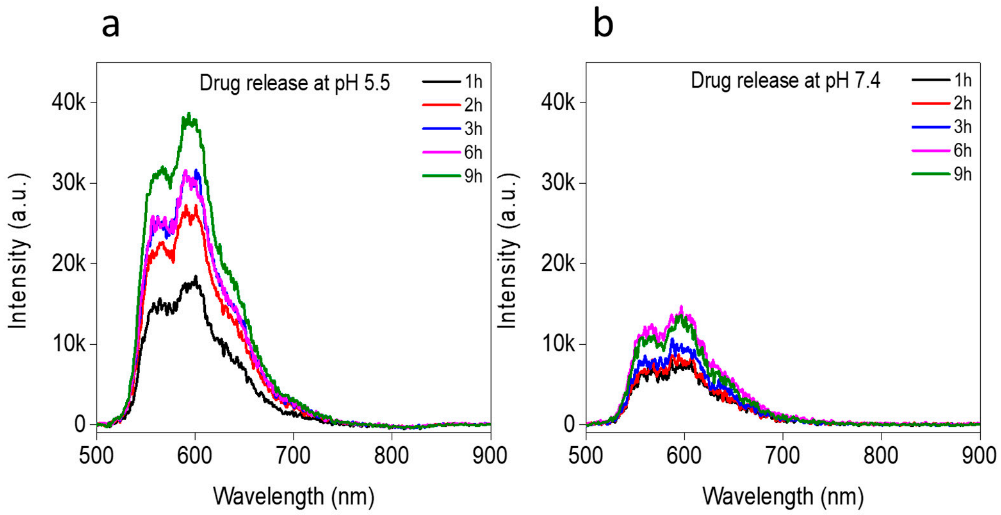

Figure S5. Fluorescence spectra of DOX-BPWS release at (**a**) pH 5.5 (**b**) pH 7.4.

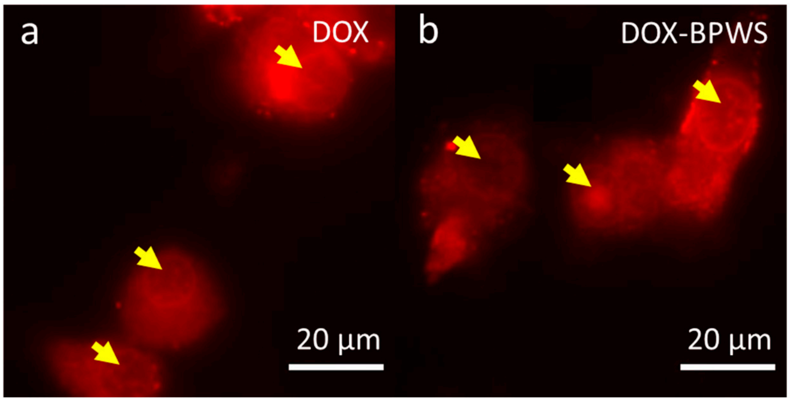

Figure S6. Fluorescence microscope images (DOX emission) of MCF-7 breast cancer cells after 24 hours. (**a**) Free DOX, (**b**) DOX-BPWS; Yellow arrow: nucleus.

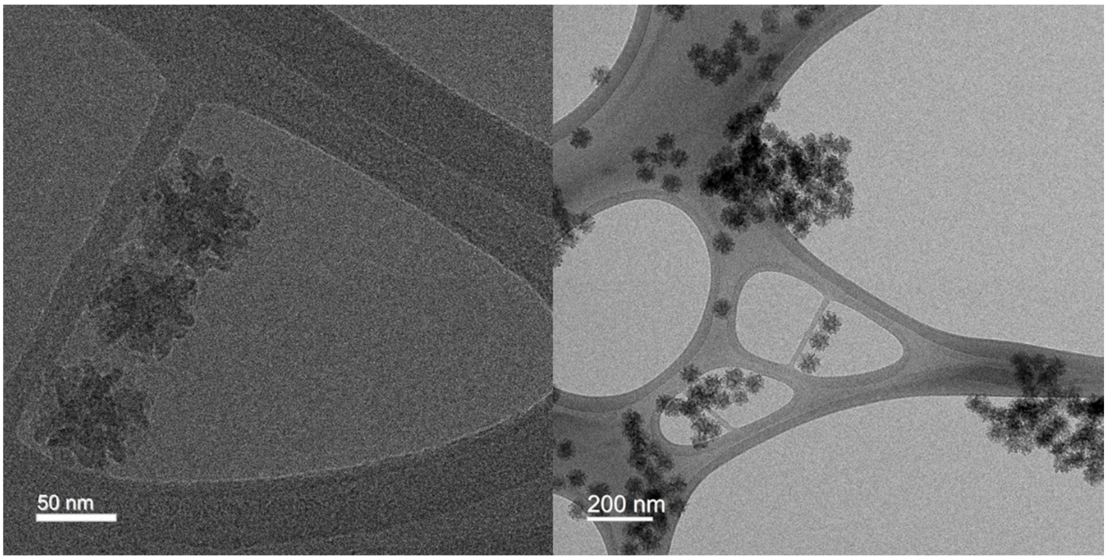

Figure S7. TEM images of FITC-BPWS showing the BPWS particle size and morphology after FITC modification.

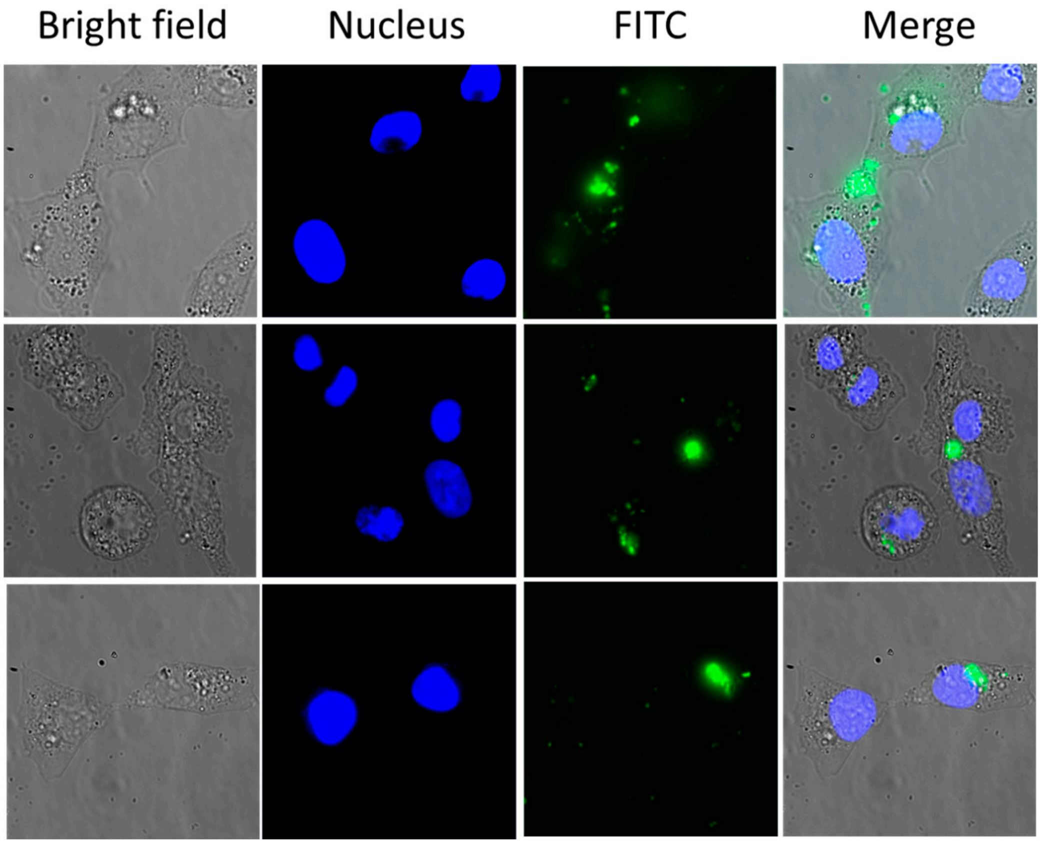

Figure S8. Fluorescence images of cancer cell permeability and intracellular uptake behaviors.

For calculation:

4,4′-bis(triethoxysilyl)-1,1′-biphenyl (BP) molecular weight = 478.73 g/mole

tetraethyl orthosilicate (TEOS) molecular weight = 208.33 g/mole

478.73 g/mole * 0.6 mmole = 287.23 mg

208.33 g/mole * 2.4 mmole = 499.99 mg

287.23 mg + 499.99 mg = 787.222 mg

287.22 mg / 787.22 mg = 0.36 (36%)
